# Crimean–Congo Haemorrhagic Fever (CCHF) in animals: Global characterization and evolution from 2006 to 2019

**DOI:** 10.1111/tbed.14120

**Published:** 2021-05-09

**Authors:** Angela Fanelli, Paolo Tizzani, Domenico Buonavoglia

**Affiliations:** ^1^ Department of Veterinary Medicine University of Bari Valenzano, Bari Italy; ^2^ Department of Veterinary Sciences University of Turin Grugliasco, Turin Italy

**Keywords:** CCHF, Crimean–Congo haemorrhagic fever, global distribution, Information System, OIE, World Animal Health

## Abstract

This study describes the global distribution and temporal evolution of Crimean–Congo haemorrhagic fever (CCHF) during the period 2006–2019, using the information officially reported to the World Organisation for Animal Health (OIE) by the National Veterinary Services of 210 countries. Eight per cent (CI 95% 4–12) of the countries reported the disease as present at least once during the study period, whereas 82% (CI 95% 77–87) as absent. Information on CCHF status lacked for 10% (CI 95% 6–13) of the countries. The majority of the countries (46%, CI 95% 39–53) never declared CCHF as notifiable, whereas only 27% (CI 95% 21–33) reported the disease as notifiable during the large majority (≥76%) of the study period. The percentage of countries that routinely applied some CCHF control measures were as following: 14% (CI 95% 9–18) passive surveillance, 3% (CI 95% 0.9–5) active surveillance and 1% (CI 95% ‐0.2–3) control of vector. The time series analysis indicates a significant decreasing trend in the percentage of countries reporting no information, whereas the percentage of countries applying disease control measures significantly increase. This finding may reflect the increased awareness on the importance of the disease and the increased efforts to monitor virus circulation in animals through the application of surveillance programmes. Out of 25 countries reporting cases in humans since 2006, only 12 report cases in animals, pointing out the lack of surveillance capacity in animal populations for some countries. The paucity of CCHF notifications in animals may also reflect the difficulty in identifying the infection due to absent or mild clinical signs. Given that the implementation of surveillance programmes by the Veterinary Services is an essential tool for monitoring the virus circulation and prevent its further spread, National Veterinary Services should keep monitoring and reporting information on CCHF, and at the same time, they should improve the quality and accuracy of the information provided.

## 1 INTRODUCTION

Crimean–Congo haemorrhagic fever (CCHF) is a tick‐borne disease caused by the arbovirus Crimean–Congo haemorrhagic fever virus (CCHFV; family *Nairoviridae*). The virus name derived from the geographic areas where it was firstly identified; in the Crimea region of the former Soviet Union in 1944 and in the Belgian Congo (currently named Democratic Republic of the Congo, DRC) in 1956 (Bente et al., 2013). Nowadays, the disease has been recognized as being endemic or potentially endemic in about 50 countries throughout Europe, Africa and Asia (Nasirian, 2020). CCHF is considered the most important tick‐borne viral disease of humans, causing severe illness characterized by fever, weakness, myalgia and haemorrhagic signs (Whitehouse, 2004). Its lethality rate ranges from 5% to 80% (Yen et al., 1985; Yilmaz et al., 2008), and there is currently no approved vaccine or specific antiviral therapy for CCHF (Keshtkar‐Jahromi et al., 2011).

The natural cycle of CCHFV includes transovarial and transstadial transmission among ixodid ticks and a cycle involving different wild and domestic vertebrates. Animals, in contrast to humans, do not show signs of illness. The role of animals, acting as reservoir of the virus, has been highlighted by several authors that reported the presence of asymptomatic viremia lasting up to 7–15 days (Ergönül, 2006; Whitehouse, 2004). Some bird species seem refractory to develop CCHF viremia; however, their role in the epidemiology of the disease is still unclear. Ground‐feeding birds appear particularly important in the ecology and epizootiology of CCHF by transporting potentially virus‐infected ticks (Whitehouse, 2004). The major route of infection for humans is represented by the bites of infected ticks, but also by the exposure to the blood of infected wild or domestic animals. In endemic regions, cases of people acquiring the infection through the contact and consumption of raw fresh or under‐cooked meat immediately after slaughtering have been described (Fazlalipour et al., 2016; Mostafavi et al., 2017). Human‐to‐human transmission through close contacts or nosocomial infections has been documented as well (Garrison et al., 2019).

Although a number of tick genera can be infected with CCHFV, ticks of the genus *Hyalomma* are considered the most important in the epidemiology of the disease, with the distribution of human cases mirroring *Hyalomma* distribution (Spengler et al., 2016).

Over the last years, climatic and environmental changes, as well as the increasing global trade and mobility, are affecting the epidemiology of CCHF, representing a threat for the further spread of the disease. As a consequence, CCHF has captured the public attention, with the increased interest of the international organizations to foster global surveillance. Indeed, CCHF is identified as a priority disease within the Global Early Warning and Response System (GLEWS), a network composed by the World Organization for Animal Health (OIE), the Food and Agriculture Organization (FAO) and the World Health Organization (WHO) with the main objective to improve detection of health threats and events of potential concern at the human–animal–ecosystem interface (http://www.glews.net/). Additionally, since 2005 (implemented in 2006), the disease is listed in the OIE list of notifiable diseases (World Organisation for Animal Health, 2020a), with the legal obligation of Member Countries to report the occurrence of CCHF.

Extensive research has been conducted on CCHFV infection in both animals and humans, including two recent systematic reviews and meta‐analysis (Nasirian, 2019, 2020). According to the scientific literature, CCHF infections in animals have been described in countries which have not officially reported data to the OIE. Considering that official information on disease distribution and epidemiological situation is of pivotal importance for disease control and prevention, this study uses data officially reported by the National Veterinary Services to the OIE to provide a comprehensive assessment of CCHF epidemiological situation at global scale.

## 2 MATERIAL AND METHODS

Data used in this study were retrieved from the OIE database: the World Animal Health Information System (WAHIS) (World Organisation for Animal Health (OIE), 2020b). This system contains information submitted by the National Veterinary Authorities of Member Countries. WAHIS is a dynamic database constantly updated, and data included in this study refer to the information available as of 3 January 2021.

### 2.1 CCHF status and control measures from 2006 to 2019

To assess the status of CCHF throughout the study period, we followed the approach described in Fanelli et al., (2020). The following time series were built: the yearly percentage of countries reporting the disease as present, absent and no information during the period 2006–2019.

In addition, we computed the yearly percentage of countries reporting:

- the obligation of disease notification
- the implementation of active surveillance
- the implementation of passive surveillance
- the implementation of control of vectors

All the percentages have been computed over the number of years for which the countries reported information.

All the time series were then formatted into a time series object using the ts () function in the R software 3.5.2 (R Core Team, 2018). The Sen's method was used to determine whether there was a positive or negative trend in the data with their statistical significance (Sen, 1968). This method is characterized by a large flexibility (i.e. the data does not need to conform to any particular distribution), and thus it has been widely used in time series analysis in different fields (Fanelli et al., 2021; Marques da Silva et al., 2015).

### 2.2 Relationship between CCHF status and control measures applied

The Spearman's rank correlation coefficient (significance level of α = 0.05) was calculated to measure the relationship of the yearly percentage of countries reporting the disease as present, absent and no information against the yearly percentage of countries reporting the disease as notifiable, applying active surveillance, passive surveillance, or control of vectors.

### 2.3 Countries epidemiological framework

To describe the epidemiological situation of CCHF for each country, we computed the percentage of years for which the disease has been reported as present in either domestic animals or wildlife over the number of years for which the country reported information. The same approach was used for the countries reporting the disease as absent. Additionally, countries were gathered by Region according to the OIE classification.^1^

With regard to the obligation of disease notification and the application of active and passive surveillance, the control of vector at country level, we computed for each of the country the percentage of years of positive reporting over the number of years for which the country reported information. Afterwards, the percentage values were converted into categories as specified below.

Disease notification:

- ‘Never declared’ whether the country never declared CCHF as notifiable,
- ‘Infrequently declared’ whether the country declared CCHF as notifiable during up to 25% of the study period,
- ‘Moderately declared’ whether the country declared CCHF as notifiable during 26%–50% of the study period,
- ‘Frequently declared’ whether the country declared CCHF as notifiable during 51%–75% of the study period,
- ‘Routinely declared’ whether the country declared CCHF as notifiable during 76%–100% of the study period.

Surveillance (passive and active) and control of vector:

- ‘Never Applied’ whether the country never applied the control measure,
- ‘Infrequently applied’ whether the country applied the control the control measure during up to 25% of the study period,
- ‘Moderately applied’ whether the country applied the control the control measure during 26%–50% of the study period,
- ‘Frequently applied’ whether the country applied the control the control measure during 51%–75% of the study period,
- ‘Routinely applied’ whether the country applied the control the control measure during 76%–100% of the study period,

QGIS 3.2 (QGIS Development Team, 2017) was used to map the spatial patterns of the CCHF status and control measures over the study period.

### 2.4 Human reports versus Animal reports

We retrieved the countries reporting information on CCHF cases in humans, and for each of them, the percentage of years reporting the disease occurrence was computed over the study period. These data were then compared to the reporting in animals.

## 3 RESULTS

### 3.1 CCHF status and control measures from 2006 to 2019

**FIGURE 1:**
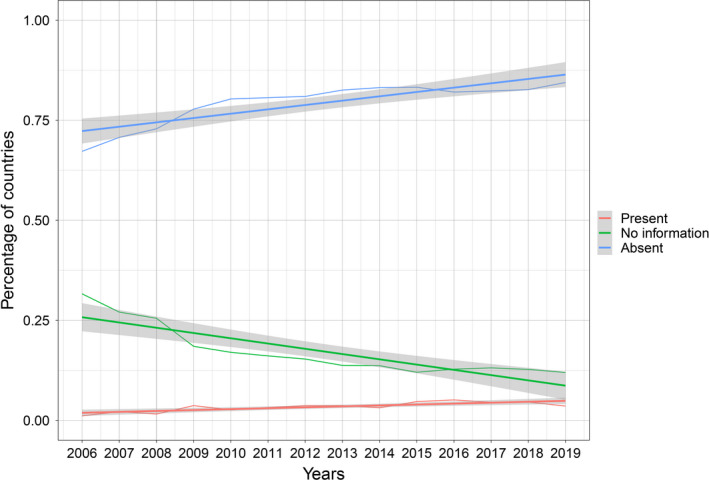
Percentage of countries reporting CCHF as present (red), absent (blue) and no information (green) from 2006 to 2019

The trend in percentage of countries reporting the disease as present absent and no information from 2006 to 2019 is shown in Figure 1. Most of the countries reported the disease as absent throughout the years, with a peak in 2019 (84%, CI 95% 79–90).

The time series analysis using Sen's method shows a significant decreasing trend in the percentage of countries reporting no information, whereas the number of countries reporting the disease as present or absent significantly increases along the period of study. Table 1 shows the results of Sen's slope estimator and the corresponding 95% confidence interval for each time series.

**TABLE 1:** Trends of time series from 2006 to 2019: percentage of countries reporting CCHF as present, percentage of countries reporting CCHF as absent, percentage of countries reporting no information

Time series (2006–2019)	Sen's slope	95% CI	*p*‐value
Percentage of countries reporting CCHF as present	0.0024	[0.001–0.0037]	*p*‐value = 0.004
Percentage of countries reporting CCHF as absent	0.0095	[0.0033–0.0148]	*p*‐value = 5.097e−05
Percentage of countries reporting no information	−0.010	[−0.016–0.006]	*p*‐value = 7.153e−06

**FIGURE 2:**
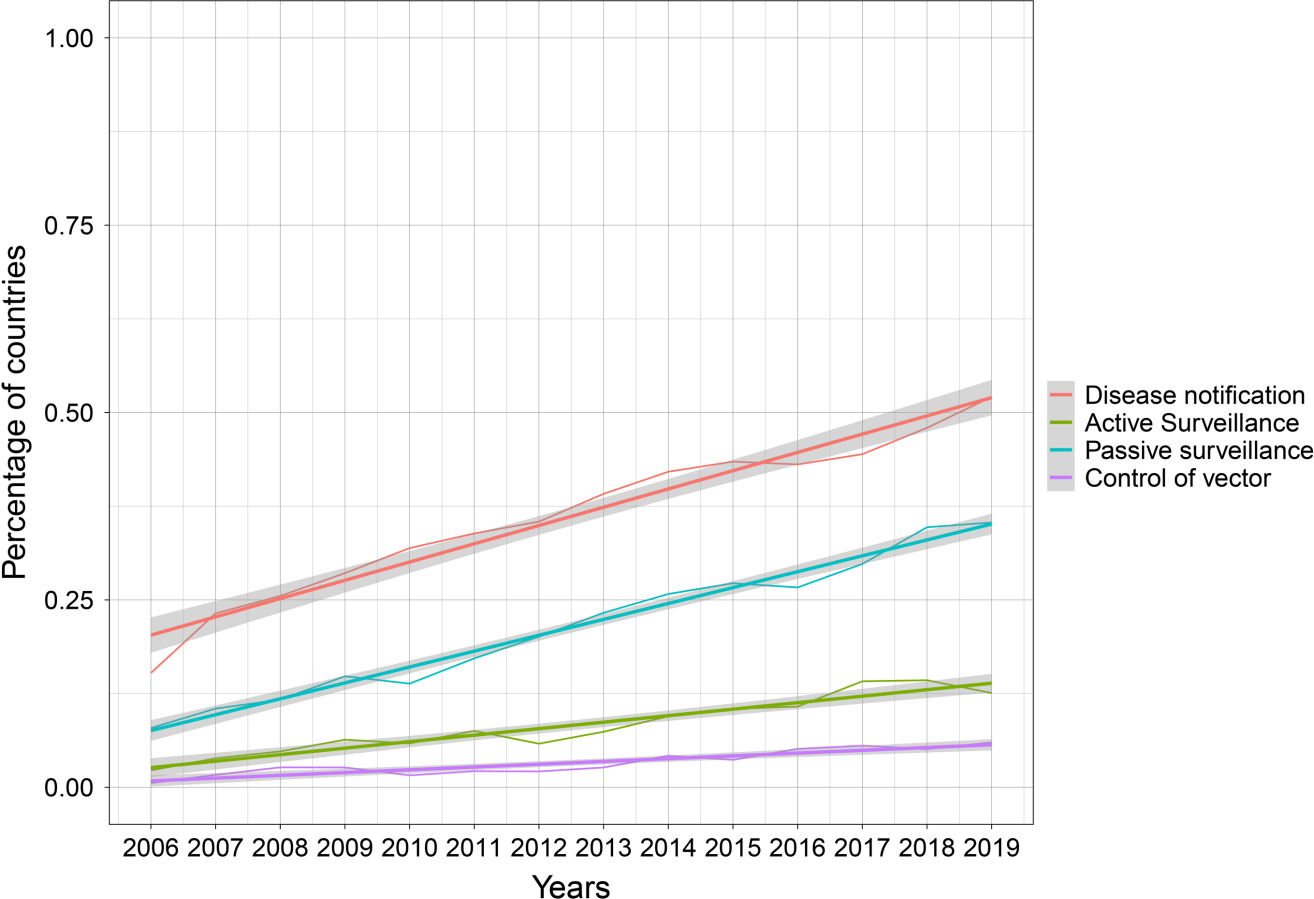
Percentage of countries declaring CCHF as notifiable (red), implementing active surveillance (green), passive surveillance (blue) and control of vector (violet) from 2006 to 2019

With regard to the obligation of disease notification and the control measures applied, there was a significant uptrend during the study period. In particular, the highest increase was observed for the obligation of disease notification, followed by the implementation of passive surveillance (Figure 2). Globally, the percentage of countries declaring the disease as notifiable rose from 15% (CI 95% 10–20) in 2006 to 52% (CI 95% 44–60) in 2019. Table 2 shows the results of Sen's slope estimator and corresponding 95% confidence interval for each time series.

**TABLE 2:** Trends of time series from 2006 to 2019: percentage of countries reporting CCHF as notifiable, percentage of countries applying active surveillance, percentage of countries applying passive surveillance, percentage of countries applying control of vector

Time series (2006–2019)	Sen's slope	95%CI	*p*‐value
Percentage of countries reporting CCHF as notifiable	0.024	[0.021–0.027]	*p*‐value = 1.453e−06
Percentage of countries applying active surveillance	0.0088	[0.0065–0.0105]	*p*‐value = 3.174e−05
Percentage of countries applying passive surveillance	0.021	[0.019–0.023]	*p*‐value = 2.501e−06
Percentage of countries applying control of vector	0.0038	[0.0026–0.0049]	*p*‐value = 0.0005

### 3.2 Relationship between CCHF status and the control measures applied

**FIGURE 3:**
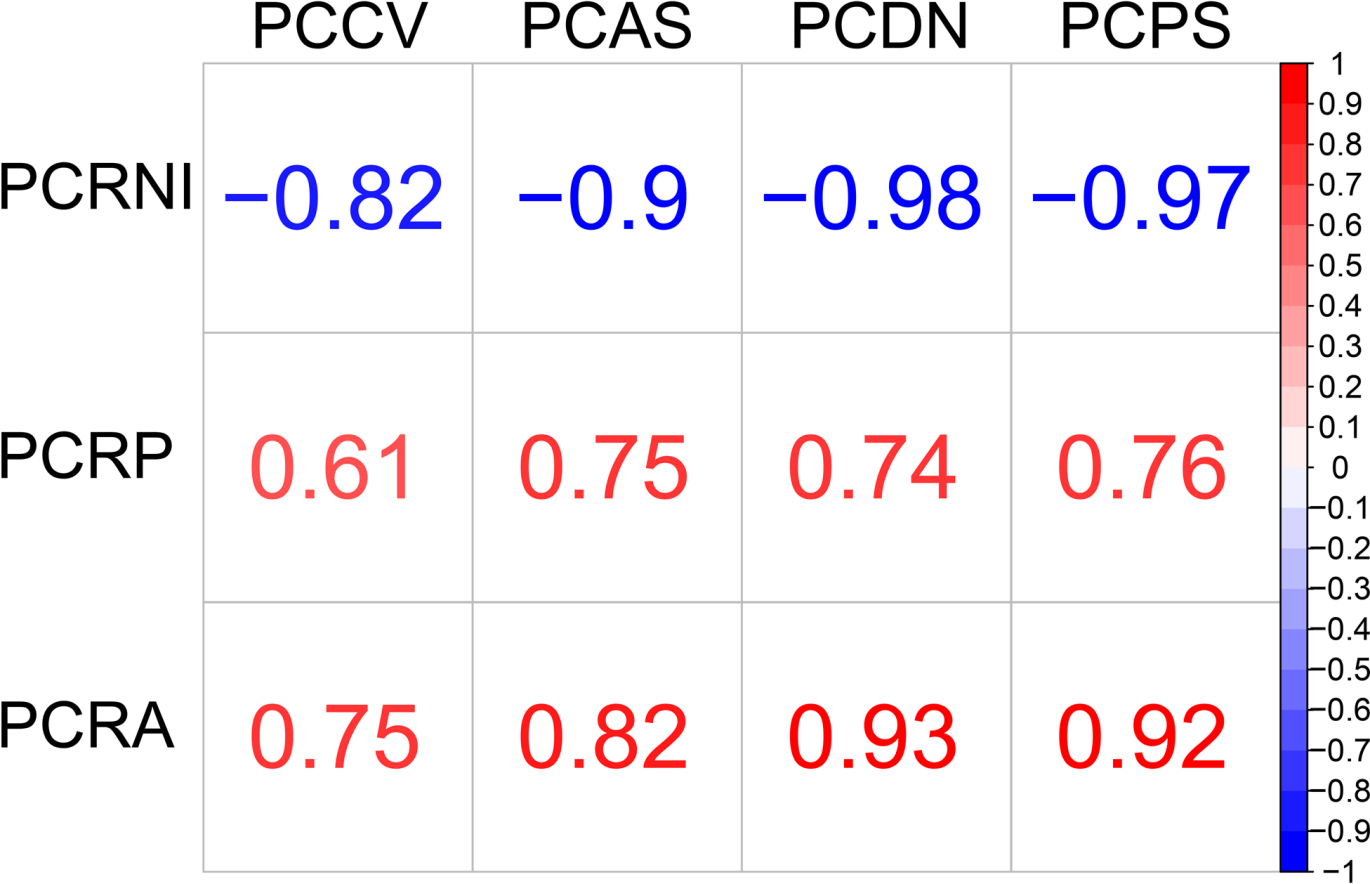
The Spearman's correlation coefficient (ρ) between: percentage of countries applying control of vector from 2006 to 2019 (PCCV), percentage of countries applying active surveillance from 2006 to 2019 (PCAS), percentage of countries declaring the disease as notifiable from 2006 to 2019 (PCDN), percentage of countries applying passive surveillance from 2006 to 2019 (PCPS), percentage of countries reporting no information from 2006 to 2019 (PCRNI), percentage of countries reporting CCHF as present from 2006 to 2019 (PCRP), percentage of countries reporting CCHF as absent from 2006 to 2019 (PCRA)

The Spearman's rank correlation coefficient indicates a statistically significant positive relationship (*p*‐value < 0.005) between the percentage of countries reporting the disease as present and absent during the study period and the different control measures applied, while there was a statistically significant negative relationship (*p*‐value < 0.005) with the percentage of countries reporting no information. As shown in Figure 3, the Spearman correlation coefficient (ρ) indicates strong relationship (ρ > |0.50|) for each pair of variables.

### 3.3 Countries epidemiological framework

**FIGURE 4:**
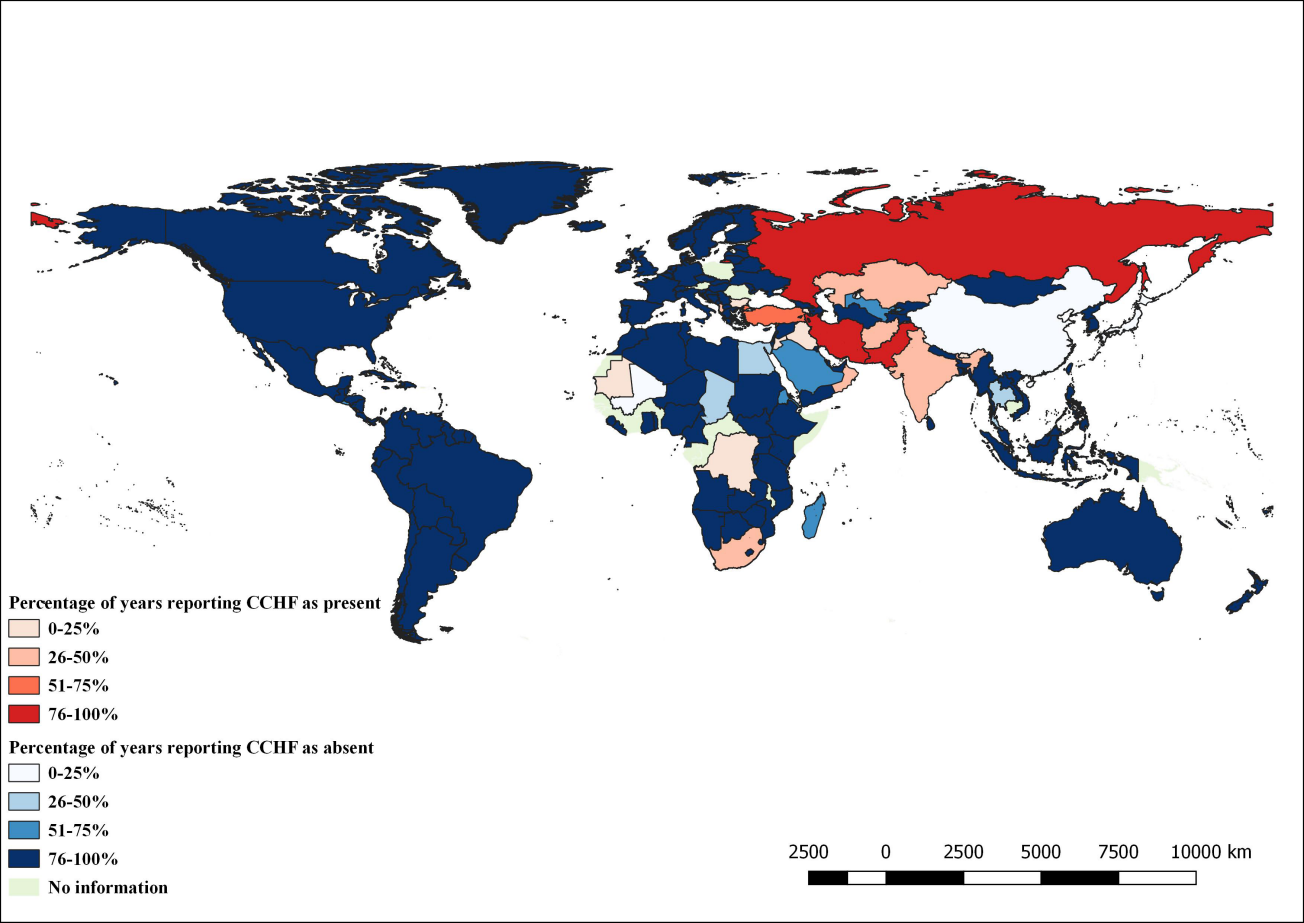
CCHF status reported by countries to the OIE from 2006 to 2019

Overall, 8% (CI 95% 4–12) of the countries reported the disease as present at least once during the study period. Among them, 41% (CI 95% 18–64) reported the disease as present during up to 25% of the study period, 35% (CI 95% 13–58) between 26% and 50%% of the study period, 6% (CI 95% ‐5–17) between 51%–75%% of the study period and 18% (CI 95% ‐0.4–36) during 76%–100% of the study period. In particular, Turkey was the only country classified in the second‐last category, whereas Iran, Pakistan and Russia were in the last one. Eighty‐two per cent (CI 95% 77–87) of the countries reported the disease as absent, with the majority of them (91%, CI 95% 86–95) reporting during 76%–100% of the study period. Information on CCHF status lacked for 10% (CI 95% 6–13) of the countries, with the majority of them localized in Africa (13 out of 20 countries). Details on CCHF status per each country are shown in Figure 4.

**FIGURE 5:**
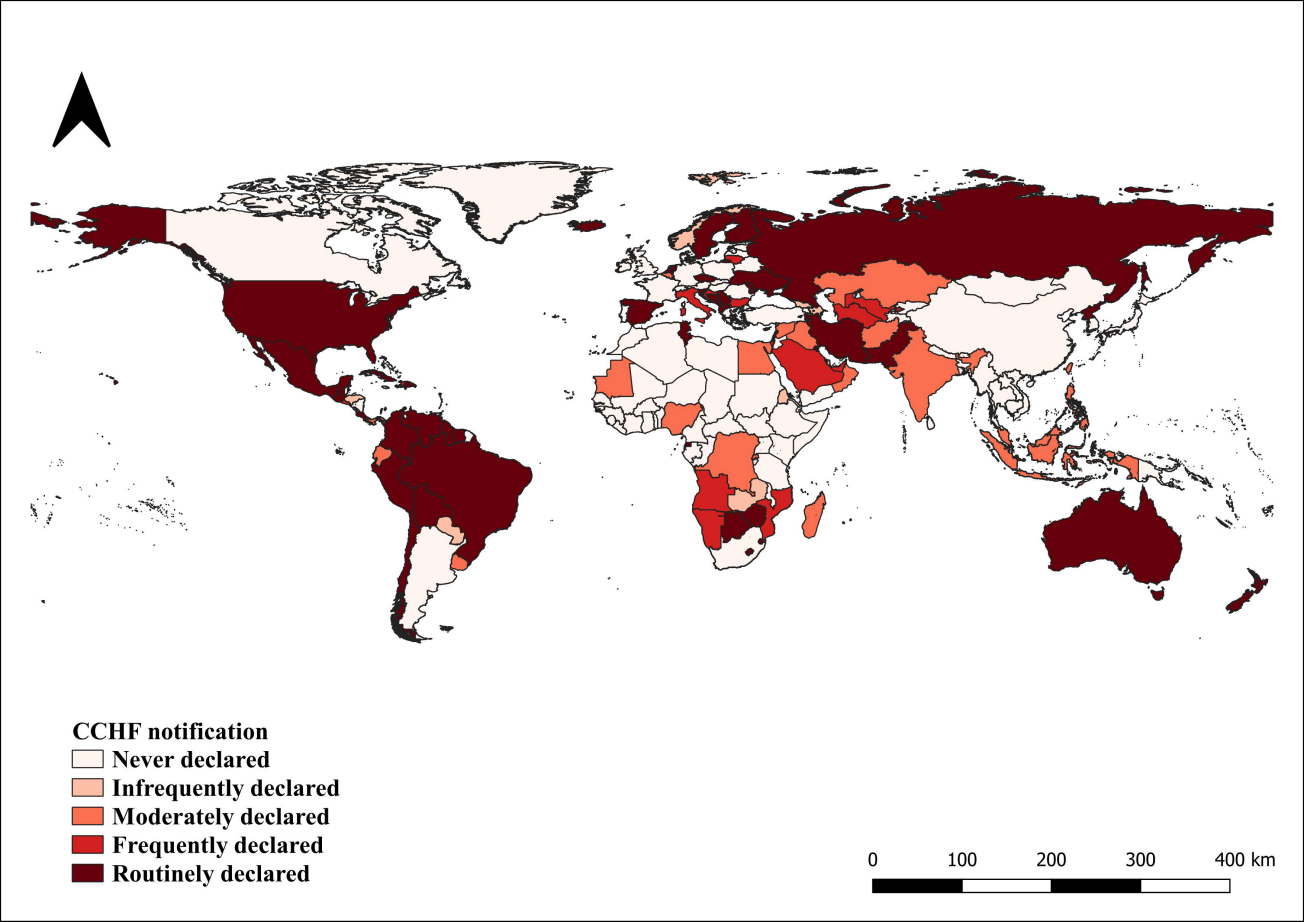
Countries reporting CCHF as notifiable disease during the study period (2006–2019)

Figure 5 shows that the majority of the countries (46%, CI 95% 39–53) never declared CCHF as notifiable, whereas only 27% (CI 95% 21–33) of the countries reported the disease as notifiable during ≥76% of the study period (‘Routinely declared’). As for the remaining countries 7% (CI 95% 4–11) are classified as ‘Infrequently declared’, 12% (CI 95% 8–16) as ‘Moderately declared’, 8% (CI 95% 4–12) as ‘Frequently declared’.

Information at Regional level on CCHF status and disease notification is reported in Table 3.

**TABLE 3:** Regional framework: percentage of countries (no of countries) reporting CCHF status and the obligation of disease notification from 2006 to 2019

Region	No countries	Disease status			Disease notification				
		Present	Absent	No info	Never declared	Infrequently declared	Moderately declared	Frequently declared	Routinely declared
Africa	59	0.05 (3)	0.74 (44)	0.20 (12)	0.591 (35)	0.07 (4)	0.13 (8)	0.051 (3)	0.15 (9)
Americas	40	0	0.97 (39)	0.02 (1)	0.27 (11)	0.12 (5)	0.1 (4)	0.05 (2)	0.45 (18)
Asia	51	0.2 (11)	0.74 (38)	0.039 (2)	0.45 (23)	0.078 (4)	0.21 (11)	0.14 (7)	0.12 (6)
Europe	44	0.07 (3)	0.86 (38)	0.068 (3)	0.48 (21)	0.045 (2)	0.02 (1)	0.09 (4)	0.36 (16)
Oceania	16	0	0.87 (14)	0.12 (2)	0.44 (7)	0	0.06 (1)	0.06 (1)	0.44 (7)

**FIGURE 6:**
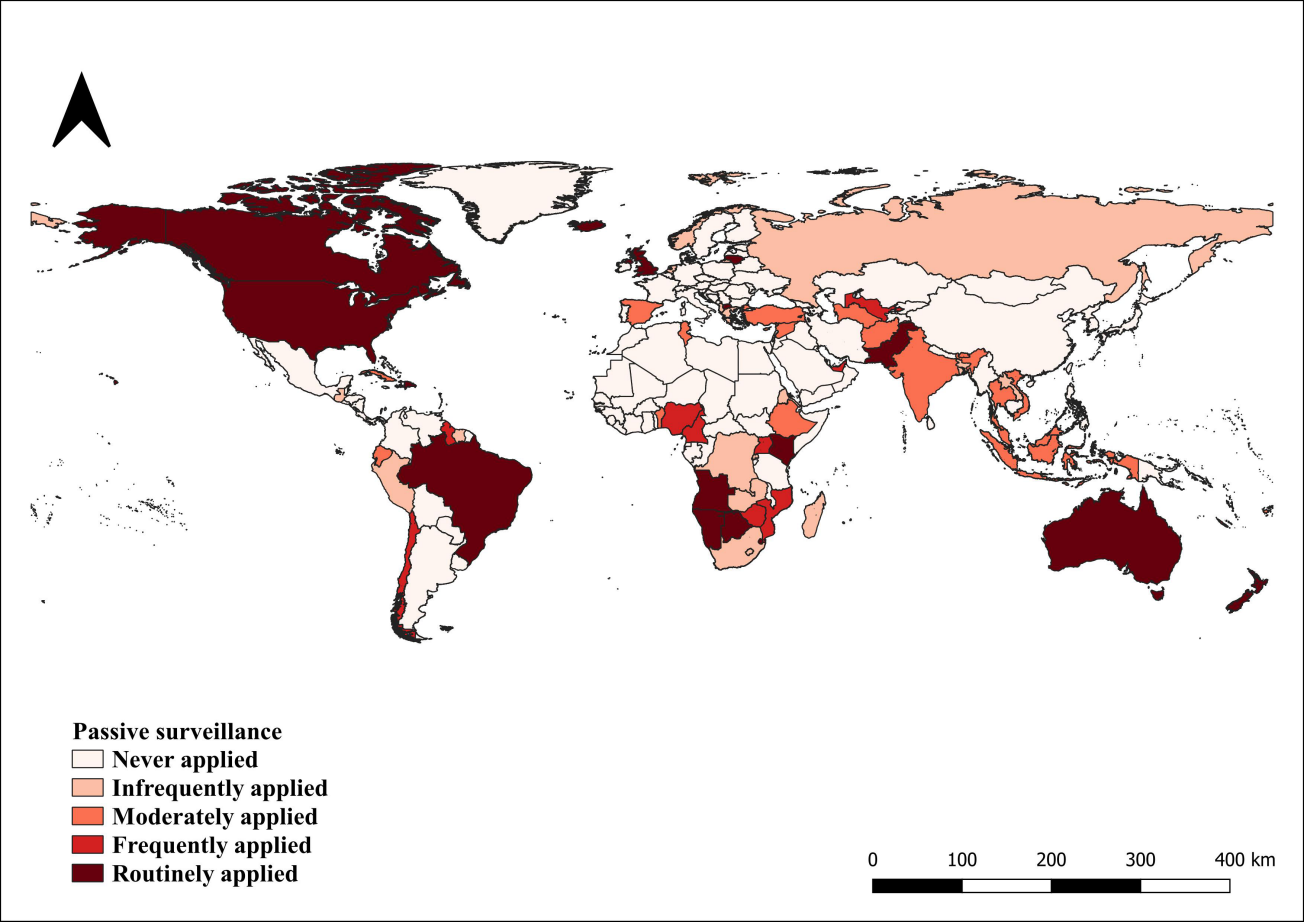
Countries reporting the application of passive surveillance during the study period (2006–2019)

With regard to the control measures, 60% (CI 95% 53–66) of the countries never applied passive surveillance, 10% (CI 95% 6–14) were classified as ‘Infrequently applied’, 9% (CI 95% 5–13) as ‘Moderately applied’, 7% (CI 95% 4–11) as ‘Frequently applied’ and 14% (CI 95% 9–18) as ‘Routinely applied’ (Figure 6).

**FIGURE 7:**
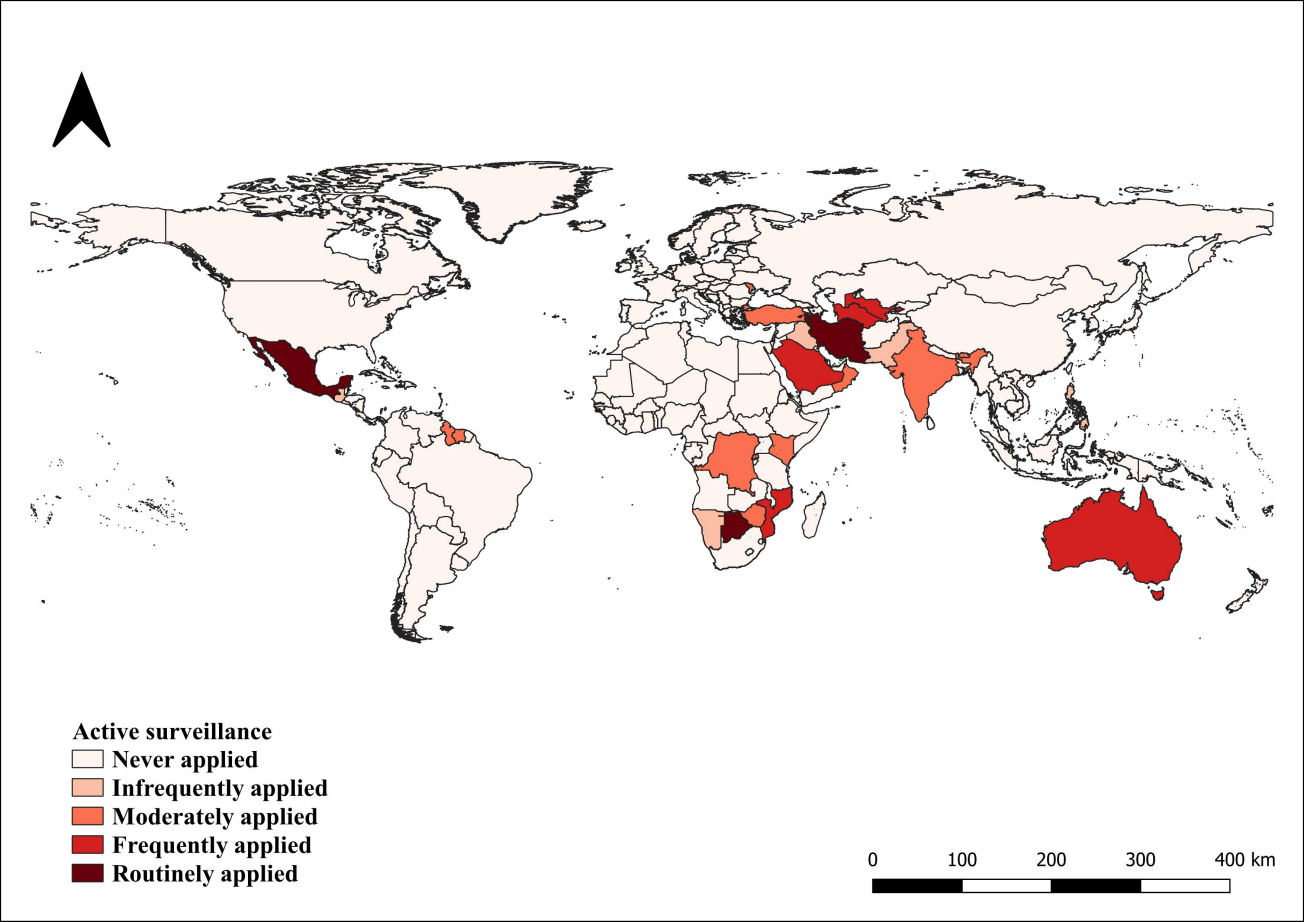
Countries reporting the application of active surveillance during the study period (2006–2019)

Also in the case of active surveillance, the majority of the countries (82%, CI 95% 77–87) fell within the category of ‘Never applied’. Four per cent (CI95 %1–7) of the countries within ‘Infrequently applied’, 7% (CI 95% 3–10) within ‘Moderately applied’, 3% (CI 95% 0.9–5) within ‘Frequently applied’ and 3% (CI 95% 0.9–5) within ‘Routinely applied’ (Figure 7).

**FIGURE 8:**
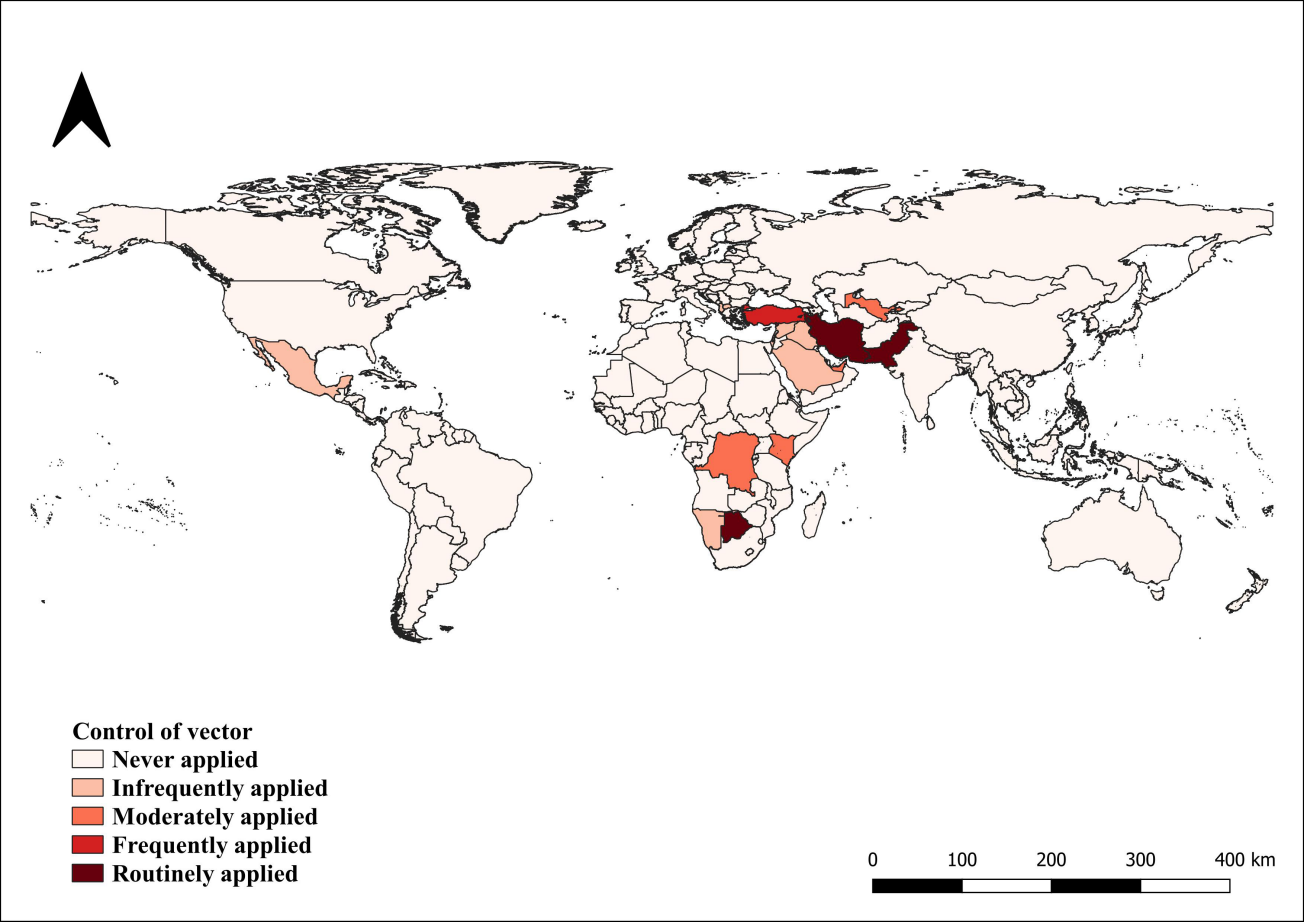
Countries reporting the application of vector control during the study period (2006–2019)

In relation to the control of vector, 92% (CI 95% 89–96) of the countries never implemented this measure. For the remaining countries; 4% (CI 95% 1–6) were classified as ‘Infrequently applied’, 2% (CI 95% 0.05–4) as ‘Moderately applied’, only 1 countries (0.5%, CI 95% ‐0.4–1) as ‘Frequently applied’ and 3 countries (1%, CI 95% ‐0.2–3) as ‘Routinely applied’ (Figure 8). The last two categories included Turkey, applying control of vector during 51 to 75% of the study period, and Botswana, Pakistan and Iran which applied this measure during 76%–100% of the study period.

Information at regional level on CCHF control measures is reported in Table 4.

**TABLE 4:** Regional framework: percentage of countries (n° of countries) reporting control measures for CCHF from 2006 to 2019

Region		Africa	Americas	Asia	Europe	Oceania
No countries		59	40	51	44	16
Passive surveillance	Never applied	0.56 (33)	0.6 (24)	0.65 (33)	0.75 (33)	0.125 (2)
	Infrequently applied	0.13 (8)	0.07 (3)	0.06 (3)	0.14 (6)	0.06 (1)
	Moderately applied	0.07 (4)	0.05 (2)	0.21 (11)	0.023 (1)	0.12 (2)
	Frequently applied	0.10 (6)	0.07 (3)	0.06 (3)	0	0.19 (3)
	Routinely applied	0.13 (8)	0.2 (8)	0.02 (1)	0.09 (4)	0.5 (8)
Active surveillance	Never applied	0.85 (50)	0.85 (34)	0.76 (39)	0.98 (43)	0.44 (7)
	Infrequently applied	0.03 (2)	0.02 (1)	0.08 (4)	0	0.12 (2)
	Moderately applied	0.07 (4)	0.07 (3)	0.08 (4)	0.02 (1)	0.1 (2)
	Frequently applied	0.02 (1)	0	0.06 (3)	0	0.19 (3)
	Routinely applied	0.03 (2)	0.05 (2)	0.02 (1)	0	0.12 (2)
Control vector	Never applied	0.93 (55)	0.97 (39)	0.82 (42)	0.95 (42)	1 (16)
	Infrequently applied	0.017 (1)	0.02 (1)	0.08 (4)	0.04 (2)	0
	Moderately applied	0.03 (2)	0	0.04 (2)	0	0
	Frequently applied	0	0	0.02 (1)	0	0
	Routinely applied	0.02 (1)	0	0.04 (2)	0	0

### 3.4 Human reports versus Animal reports

Twenty‐five countries reported human cases to the OIE during the study period. For each country, the comparison between the percentage of years of CCHF reporting in humans and in animals is drawn by Table 5.

**TABLE 5:** Comparison between the percentage of years reporting CCHF in humans and animals

Country	Percentage years reporting CCHF in humans	CCHF status in animals	Percentage years reporting CCHF in animals
Afghanistan	0.43	Present	0.29
Albania	0.07	Present	0.36
Bulgaria	0.57	Present	0.07
Central African Republic	0.07	No info	n.a.
China	0.07	Absent	0.14
Spain	0.14	Absent	1.00
United Kingdom	0.07	Absent	0.93
Georgia	0.57	Absent	1.00
Guinea	0.07	No info	n.a.
Greece	0.07	Absent	1.00
India	0.50	Present	0.43
Iran	0.71	Present	0.93
Iraq	0.29	Present	0.07
Kazakhstan	0.36	Present	0.36
North Macedonia	0.07	Absent	1.00
Namibia	0.21	Absent	0.93
Oman	0.50	Present	0.50
Pakistan	0.50	Present	0.93
Russia	0.86	Present	1.00
Senegal	0.21	No info	n.a.
Serbia	0.07	Absent	1.00
Tajikistan	0.07	Absent	0.92
Turkey	1.00	Present	0.64
Uganda	0.14	Absent	1.00
South Africa	0.93	Present	0.36

Abbreviation: n.a., not applicable.

## 4 DISCUSSION

This paper describes the global distribution and temporal evolution of CCHF in animals during a 14‐year period (2006–2019) using the information officially reported to the OIE by the National Veterinary Services of 210 countries. Results from this study provide a picture of CCHF status and evolution worldwide, highlighting a significant increase in the number of countries reporting information on the disease. This finding is in line with the uptrend of CCHF cases observed in humans through the past decades (Nasirian, 2020). Most probably this observation reflects an increased global awareness on the importance of CCHF rather than a deterioration of the epidemiological situation of the disease.

In endemic regions such as Africa and Asia (Spengler et al., 2019), the percentages of countries reporting no information decreased from 60% to 27% and 32% to 10%, respectively, throughout the study period.

The reduction of the percentage of countries reporting no information may also be due to the improved efforts to monitor the virus circulation in animals through specific surveillance programmes. In fact, the rise of reports indicating CCHFV circulation in humans in some regions was linked with the implementation of surveillance systems, suggesting that the virus was already present in the area (Greiner et al., 2015). The Spearman's test confirms the significant impact of the application of control measures (and the obligation of disease notification) and the increase of the information reported on CCHF.

In agreement with the rise of reporting, significant increasing trends were detected for the obligation of disease notification at country level as well as for the implementation of specific measures related to disease monitoring and control. Specifically, the rise in time of the application of passive surveillance was substantial compared to the increase in the application of active surveillance and control of vector. Considering the epidemiological characteristics of the disease, that is mainly asymptomatic in animals (Spengler et al., 2016), the application of active surveillance is the main approach improving the probability of detection of the virus.

With regard to the geographic distribution, most of the countries reporting the disease as present are localized in Asia and to a minor extent in Africa. Indeed, the recognized geographic distribution of CCHFV includes several areas: Asia, Eastern Europe, Middle East and most of Africa (Spengler et al., 2019). Nevertheless, there are discrepancies between the official data reported to the OIE and the information available in the literature on CCHF. Several studies were published on the occurrence of CCHF infection in animals, including a review summarizing reports of the presence or absence of CCHFV antibodies in domestic and wild populations (Spengler et al., 2016). When comparing the information of the literature with the results of this study, it is noticeable that in Europe the virus is circulating not only in Bulgaria and Albania, as reported by the National Veterinary Services to the OIE, but also in Hungary, Romania and Spain (Ruiz‐Fons et al., 2018; Spengler et al., 2016). Similarly, in Africa the countries reporting CCHF as present to the OIE are fewer than the ones where scientific studies demonstrated the virus circulation in animals. On the contrary, the Democratic Republic of the Congo reported CCHF as present during up to 25% of the study period, while no scientific study on animal population in the country is available. The differences between the official data and the scientific literature may be explained within the context of the difficulties of the veterinary services in conducting accurate surveillance on animal populations at country level. Critical constrains in disease surveillance are mainly related to the costs of support/expertise, resources/infrastructure and training of the National Veterinary Services. These conditions impair the implementation of data collection and proper diagnostic. On this matter, the disease status reported by the countries appears to be in accordance with the level of surveillance activities declared. In fact, only few countries reported the application of active surveillance, that is the main measure that allows to detect the circulation of the virus in asymptomatic animal populations.

Another indicator of the capacity of the veterinary services to detect the virus circulation is represented by the comparison of the reporting of CCHF in humans and animals. Out to 25 countries reporting cases in humans since 2006 only 12 report cases in animals as well. Considering the epidemiology of the disease, this discrepancy stresses the lack of surveillance capacity in animal populations in some countries with active viral circulation.

## 5 CONCLUSIONS

This study aims to provide a comprehensive overview on CCHF status and evolution worldwide. This is also one of the few available studies that uses official information reported by the National Veterinary Services to the OIE (Cárdenas et al., 2019, Fanelli et al., 2020, Fanelli & Tizzani, 2020). A precise picture representing the historical and current CCHF data at global level can help to understand the evolution of the disease as well as improve surveillance and control programmes, resulting very useful in health policy planning.

Crimean–Congo haemorrhagic fever virus is circulating in animal populations of several countries. The implementation of surveillance programmes by the National Veterinary Services is an essential tool for monitoring the level transmission and presence and for investigating areas where viral circulation is not known. In conclusion, this work highlights the importance of having a global data set collecting information on disease status and evolution. The data collected in WAHIS need to be kept updated and the quality of the information reported increased, as much as possible, to serve the global efforts for CCHF control and prevention.

## CONFLICT OF INTEREST

The authors declare that the research was conducted in the absence of any commercial or financial relationships that could be construed as a potential conflict of interest.

## Supporting information

Supplementary Material

## Data Availability

The data that support the findings of this study are available on the World Animal Health Information Database (WAHIS) Interface: https://wahis.oie.int

## ENDNOTE

^1^

https://www.oie.int/about‐us/wo/oie‐regional‐commissions/
