# Supplementary material for: Crimean–Congo Haemorrhagic Fever (CCHF) in animals: Global characterization and evolution from 2006 to 2019
Source: Transbound Emerg Dis. 2021 May 9;69(3):1556–67. doi: 10.1111/tbed.14120 (PMC9291194; doi:10.1111/tbed.14120)
Supplement: Supplementary file 1 — Supplementary Material [file TBED-69-1556-s001.docx]

**Table S1:** Comparison between the reports of CCHF occurrence in scientific literature and notification from National Veterinary Services.

| **Country** | **OIE-status** | **Percentage of years reporting CCHF to OIE (2006-2019)** | **Detection of anti-CCHFV antibodies (Nasirian 2019)** |
| --- | --- | --- | --- |
| Afghanistan | Present | 26-50% | Reported |
| Albania | Present | 26-50% | Reported |
| Algeria | Absent | 76-100% | No information |
| Andorra | Absent | 76-100% | No information |
| Angola | Absent | 76-100% | No information |
| Argentina | Absent | 76-100% | No information |
| Armenia | Present | 1-25% | Reported prior to 2006 |
| Aruba | No information | n.a. | No information |
| Australia | Absent | 76-100% | No information |
| Austria | No information | n.a. | No information |
| Azerbaijan | Absent | 76-100% | Reported prior to 2006 |
| Bahamas | Absent | 76-100% | No information |
| Bahrain | Absent | 76-100% | No information |
| Bangladesh | Absent | 76-100% | No information |
| Barbados | Absent | 76-100% | No information |
| Belarus | Absent | 76-100% | No information |
| Belgium | Absent | 76-100% | No information |
| Belize | Absent | 76-100% | No information |
| Benin | No information | n.a. | No information |
| Bhutan | Present | 1-25% | No information |
| Bolivia | Absent | 76-100% | No information |
| Bosnia and Herzegovina | Absent | 76-100% | No information |
| Botswana | Absent | 76-100% | No information |
| Brazil | Absent | 76-100% | No information |
| Brunei Darussalam | Absent | 76-100% | No information |
| Bulgaria | Present | 1-25% | Reported |
| Burkina Faso | No information | n.a. | No information |
| Burundi | Absent | 76-100% | No information |
| Cambodia | No information | n.a. | No information |
| Cameroon | Absent | 76-100% | No information |
| Canada | Absent | 76-100% | No information |
| Cape Verde | Absent | 76-100% | No information |
| Cayman Islands | Absent | 76-100% | No information |
| Central African Republic | No information | n.a. | No information |
| Ceuta | Absent | 76-100% | No information |
| Chad | Absent | 26-50% | No information |
| Chile | Absent | 76-100% | No information |
| China (People's Rep. of) | Absent | 1-25% | Reported prior to 2006 |
| Chinese Taipei | Absent | 76-100% | No information |
| Colombia | Absent | 76-100% | No information |
| Comoros | Absent | 76-100% | No information |
| Congo (Dem. Rep. of the) | Present | 1-25% | No information |
| Congo (Rep. of the) | No information | n.a. | No information |
| Cook Islands | Absent | 76-100% | No information |
| Costa Rica | Absent | 76-100% | No information |
| Cote D'Ivoire | No information | n.a. | No information |
| Croatia | Absent | 76-100% | No information |
| Cuba | Absent | 76-100% | No information |
| Cyprus | Absent | 76-100% | No information |
| Czech Republic | Absent | 76-100% | No information |
| Denmark | Absent | 76-100% | No information |
| Djibouti | Absent | 1-25% | No information |
| Dominica | Absent | 76-100% | No information |
| Dominican Republic | Absent | 76-100% | No information |
| Ecuador | Absent | 76-100% | No information |
| Egypt | Absent | 26-50% | Reported |
| El Salvador | Absent | 51-75% | No information |
| Equatorial Guinea | Absent | 76-100% | No information |
| Eritrea | Absent | 51-75% | No information |
| Estonia | Absent | 76-100% | No information |
| Ethiopia | Absent | 76-100% | No information |
| Faeroe Islands | Absent | 76-100% | No information |
| Falkland Islands (Malvinas) | Absent | 76-100% | No information |
| Fiji | Absent | 76-100% | No information |
| Finland | Absent | 76-100% | No information |
| France | Absent | 76-100% | No information |
| French Guiana | Absent | 76-100% | No information |
| French Polynesia | Absent | 76-100% | No information |
| Gabon | No information | n.a. | No information |
| Gambia | No information | n.a. | No information |
| Georgia | Absent | 76-100% | No information |
| Germany | Absent | 76-100% | No information |
| Ghana | Absent | 76-100% | No information |
| Greece | Absent | 76-100% | Reported |
| Greenland | Absent | 76-100% | No information |
| Grenada | Absent | 76-100% | No information |
| Guadeloupe (France) | Absent | 1-25% | No information |
| Guatemala | Absent | 76-100% | No information |
| Guinea | No information | n.a. | No information |
| Guinea-Bissau | No information | n.a. | No information |
| Guyana | Absent | 76-100% | No information |
| Haiti | Absent | 76-100% | No information |
| Honduras | Absent | 76-100% | No information |
| Hong Kong (SAR - PRC) | Absent | 1-25% | No information |
| Hungary | Absent | 76-100% | Reported prior to 2006 |
| Iceland | Absent | 76-100% | No information |
| India | Present | 26-50% | Reported |
| Indonesia | Absent | 76-100% | No information |
| Iran | Present | 76-100% | Reported |
| Iraq | Present | 1-25% | Reported prior to 2006 |
| Ireland | Absent | 76-100% | Reported prior to 2006 |
| Israel | Absent | 76-100% | No information |
| Italy | Absent | 76-100% | No information |
| Jamaica | Absent | 76-100% | No information |
| Japan | Absent | 1-25% | No information |
| Jordan | Present | 1-25% | No information |
| Kazakhstan | Present | 26-50% | Reported prior to 2006 |
| Kenya | Absent | 76-100% | Reported prior to 2006 |
| Kiribati | Absent | 76-100% | No information |
| Korea (Dem. People's Rep.) | Absent | 76-100% | No information |
| Korea (Rep. of) | Absent | 76-100% | No information |
| Kuwait | Absent | 76-100% | No information |
| Kyrgyzstan | Absent | 76-100% | No information |
| Laos | Absent | 76-100% | No information |
| Latvia | Absent | 76-100% | No information |
| Lebanon | Absent | 76-100% | No information |
| Lesotho | Absent | 76-100% | No information |
| Liberia | Absent | 76-100% | No information |
| Libya | Absent | 76-100% | No information |
| Liechtenstein | Absent | 76-100% | No information |
| Lithuania | Absent | 76-100% | No information |
| Luxembourg | Absent | 76-100% | No information |
| Madagascar | Absent | 51-75% | No information |
| Malawi | No information | n.a | No information |
| Malaysia | Absent | 76-100% | No information |
| Maldives | Absent | 76-100% | No information |
| Mali | Absent | 1-25% | Reported |
| Malta | Absent | 76-100% | No information |
| Marshall Islands | Absent | 76-100% | No information |
| Martinique (France) | Absent | 76-100% | No information |
| Mauritania | Present | 1-25% | Reported |
| Mauritius | Absent | 76-100% | No information |
| Mayotte (France) | Absent | 76-100% | No information |
| Melilla | Absent | 26-50% | No information |
| Mexico | Absent | 76-100% | No information |
| Micronesia (Federated States) | Absent | 76-100% | No information |
| Moldova | Absent | 76-100% | No information |
| Mongolia | Absent | 76-100% | No information |
| Montenegro | Absent | 76-100% | No information |
| Morocco | Absent | 76-100% | No information |
| Mozambique | Absent | 76-100% | No information |
| Myanmar | Absent | 76-100% | No information |
| Namibia | Absent | 76-100% | No information |
| Nepal | Absent | 76-100% | No information |
| Netherlands | Absent | 76-100% | No information |
| New Caledonia | Absent | 76-100% | No information |
| New Zealand | Absent | 76-100% | No information |
| Nicaragua | Absent | 76-100% | No information |
| Niger | Absent | 76-100% | Reported prior to 2006 |
| Nigeria | Absent | 76-100% | Reported prior to 2006 |
| Norway | Absent | 76-100% | No information |
| Oman | Present | 26-50% | Reported prior to 2006 |
| Pakistan | Present | 76-100% | Reported prior to 2006 |
| Palau | Absent | 76-100% | No information |
| Palestinian Auton. Territories | Absent | 76-100% | No information |
| Panama | Absent | 76-100% | No information |
| Papua New Guinea | No information | n.a. | No information |
| Paraguay | Absent | 76-100% | No information |
| Peru | Absent | 76-100% | No information |
| Philippines | Absent | 76-100% | No information |
| Poland | No information | n.a. | No information |
| Portugal | Absent | 76-100% | No information |
| Qatar | Absent | 76-100% | No information |
| Rep. of Macedonia | Absent | 76-100% | Reported |
| Reunion (France) | Absent | 76-100% | No information |
| Romania | No information | n.a | Reported |
| Russia | Present | 76-100% | Reported prior to 2006 |
| Rwanda | Absent | 76-100% | No information |
| Samoa | Absent | 76-100% | No information |
| San Marino | Absent | 76-100% | No information |
| Sao Tome and Principe | Absent | 76-100% | No information |
| Saudi Arabia | Absent | 51-75% | No information |
| Senegal | No information | n.a. | Reported prior to 2006 |
| Serbia | Absent | 76-100% | No information |
| Seychelles | Absent | 76-100% | No information |
| Sierra Leone | Absent | 76-100% | No information |
| Singapore | Absent | 76-100% | No information |
| Slovakia | Absent | 76-100% | No information |
| Slovenia | Absent | 76-100% | No information |
| Solomon Islands | No information | n.a. | No information |
| Somalia | No information | n.a. | Reported prior to 2006 |
| South Africa | Present | 26-50% | Reported prior to 2006 |
| South Sudan | Absent | 76-100% | No information |
| Spain | Absent | 76-100% | No information |
| Sri Lanka | Absent | 76-100% | No information |
| St. Helena | Absent | 76-100% | No information |
| St. Lucia | Absent | 76-100% | No information |
| St. Vincent and the Grenadines | Absent | 76-100% | No information |
| Sudan | Absent | 76-100% | Reported |
| Suriname | Absent | 76-100% | No information |
| Swaziland | Absent | 76-100% | No information |
| Sweden | Absent | 76-100% | No information |
| Switzerland | Absent | 76-100% | No information |
| Syria | Absent | 76-100% | No information |
| Tajikistan | Absent | 76-100% | Reported prior to 2006 |
| Tanzania | Absent | 76-100% | Reported prior to 2006 |
| Thailand | Absent | 26-50% | No information |
| Timor-Leste | Absent | 76-100% | No information |
| Togo | Absent | 76-100% | No information |
| Tonga | Absent | 1-25% | No information |
| Trinidad and Tobago | Absent | 76-100% | No information |
| Tunisia | Absent | 76-100% | No information |
| Turkey | Present | 51-75% | Reported |
| Turkmenistan | Absent | 76-100% | Reported prior to 2006 |
| Tuvalu | No information | n.a. | No information |
| Uganda | Absent | 76-100% | Reported prior to 2006 |
| Ukraine | Absent | 76-100% | No information |
| United Arab Emirates | Absent | 76-100% | Reported prior to 2006 |
| United Kingdom | Absent | 76-100% | No information |
| United States of America | Absent | 76-100% | No information |
| Uruguay | Absent | 76-100% | No information |
| Uzbekistan | Absent | 51-75% | No information |
| Vanuatu | Absent | 76-100% | No information |
| Venezuela | Absent | 76-100% | No information |
| Vietnam | Absent | 76-100% | No information |
| Wallis and Futuna Islands | Absent | 76-100% | No information |
| Yemen | Absent | 76-100% | No information |
| Zambia | Absent | 76-100% | No information |
| Zimbabwe | Absent | 76-100% | Reported prior to 2006 |

n.a.= not applicable

Nasirian, H. (2019) Crimean-Congo hemorrhagic fever (CCHF) seroprevalence: A systematic review and meta-analysis. *Acta Tropica* 196, 102-120.
